# The Role of Horses as Instructional and Diagnostic Partners in Riding Lessons

**DOI:** 10.3390/ani15101418

**Published:** 2025-05-14

**Authors:** Beatrice Szczepek Reed, Susanne Lundesjö Kvart

**Affiliations:** 1 School of Education, Communication and Society, King’s College London, London SE1 9NH, UK; 2 Department of Animal Biosciences, Swedish University of Agricultural Sciences, 750 07 Uppsala, Sweden; susanne.lundesjo-kvart@slu.se

**Keywords:** conversation analysis, interspecies interaction, instruction, horse-riding lessons

## Abstract

In many types of embodied skills instruction, the learnables—that is, the local and jointly negotiated foci of instruction—emerge from a combination between a pre-existing lesson plan and the spontaneous interaction between teacher and student. Through the analytical lens of Conversation Analysis, this paper investigates the interspecies instruction setting of horse-riding lessons and shows how here, it is not only the human teachers and learners that determine the emergence of new learnables but also the horses. Horses’ actions can initiate new courses of action in a lesson, and horses can thus become interactional partners in the instructional project. Horse-led learnables can be initiated in at least three ways: through horses’ displays of mental or physical states that pre-date the instruction sequence; through actions that respond to local contingencies of the instruction sequence; and through actions that respond specifically to the rider’s actions. In the last case, their responses become diagnostic of the rider’s mistakes. In all three cases, the human participants take their cue from the horse and base new learnables on horses’ actions. Human-led learnables can be adjusted, changed, replaced, or abandoned completely in response to horses. The study broadens the emerging field of interspecies pragmatics to include instructional interactions involving the triad of human–human–horse triad.

## Simple Summary

This study shows that, in horse-riding lessons, riding teachers treat horses as co-teachers. We show that the human teachers’ original plans for the lesson can be influenced by horses, leading to a change of direction. For example, horses can come to the riding lesson in a certain mental or physical state. The horses’ actions that result from that state can lead riding teachers to change their instructional focus. Horses can also react to the environment and thus determine what the riding teachers focus on. Finally, horses can respond to their riders in ways that highlight riding mistakes and thus change the direction of the lesson. In all of these cases, human riding teachers take their cue from the horse and base the emerging development of the riding lesson on the horse’s actions. Human instructions can be adjusted, changed, replaced, or abandoned completely in response to horses. The study shows that horse-riding lessons are a collaborative project between humans and horses.

## 1. Introduction

The study reported here contributes to the framework of interspecies pragmatics proposed by Peltola and Simonen [1]. That is, it pursues an understanding of how interactional partners from different species “make sense of each other and achieve shared communicative goals” ([1], p. 17). Specifically, we show how horses are attributed agency in horse-riding lessons. Horse riding is a highly suitable context for an interspecies pragmatic enquiry: it involves continuous mutual attempts at sense-making, as horses try to make sense of humans and vice versa. Horse-riding *instruction* has the additional benefit that human sense-making is verbalized and thus accessible to pragmatic analysis.

Our analysis of naturally occurring horse-riding lessons reveals that horses’ actions can initiate new learning sequences, which riding teachers respond to and turn into “learnables” for riders. By “learnables” we mean the locally and jointly negotiated foci of instruction [2]. We argue that in doing so, riding teachers treat horses as instructional partners who co-teach riders how to ride. We show that riding teachers draw on three kinds of horse actions as initiating new learnables: actions that display a horse’s pre-existing inner or outer state, actions that respond directly to the local contingencies of the current instruction sequence, and actions that respond specifically to the rider’s communication with the horse. The last type is doubly relevant to a study of interspecies pragmatics. Firstly, it shows humans’ (i.e., teachers’ and riders’) verbal sense-making of horses’ actions; secondly, it shows teachers’ *verbalized interpretations of horses’ understanding* of riders’ actions.

Below, we give brief overviews of the previous literature on sequence organization in interspecies interaction and on horse riding and horse-riding instruction. We then present our data and approach before focusing on our analysis. We end with a discussion and concluding observations.

### 1.1. Sequential Organization in Interspecies Interaction

This paper contributes to the growing fields of interspecies pragmatics [1] and interspecies interaction [3,4], which are part of an increasing focus on nonhuman animals in linguistics [5,6]. Research in this area has considered human language to, for, and about animals, e.g., [7,8,9,10] as well as animals’ responses to humans’ actions, e.g., [6,11]. In addition to language use, the embodied aspects of interspecies interaction have been investigated, e.g., [12]. Horse riding involves much haptic interaction, as humans and horses communicate mostly through continuous bodily contact. Haptic interspecies interactions have been conceptualized as “coalitions of touch” [13] and as a form of “haptic sociality” [14].

Our study explores how humans make sense of and respond to horses and how horses’ responses and other actions become consequential for horse–human interaction. Importantly, our primary focus here is not equine behaviour as such but the inferences that humans make from what is observable. We are interested in humans’ interactional framing of horses’ actions (which we refer to as their “treatment” of prior actions) and the impact that horses’ actions have on humans’ subsequent actions. This mechanism of *action* –*response*–*third position response* is part of the interactional “structure” that this Special Issue mentions in its title. At the heart of such an analysis is an understanding of interaction as sequentially organized, that is, as both backward- and forward-oriented.

The most basic interactional structure is the adjacency pair [15]; for example, a yes/no question by one speaker (a First Pair Part) makes a certain kind of response from a second speaker (a Second Pair Part) normatively relevant: a *yes*, a *no*, or a reason for not being able to provide either. This structural organization has been well-described for human-only talk. Mondémé [16] shows that adjacency pairs also exist in human–animal interaction. Her study considers two sequence types, request–compliance/rejection and summons–answer. It reveals that in their interactions with humans, animals (dogs, cats, horses) can perform both First Pair Parts (requests, summons) and Second Pair Parts (compliance/rejection, answer). For example, dogs and horses can position themselves in ways that elicit strokes from humans and dogs and cats can summon humans to let them into the house. Similarly, Cornips et al. [17] show that cats can use deictic cues, such as pointing with their gaze, ears, and paws, to recruit humans to provide them with food or open doors for them. Our analysis presents another way in which animals’ actions can influence their interactions with humans. We show that through their actions, horses can prompt riding teachers to change or abandon a current teaching trajectory. In such cases, horses are treated as co-teachers and as interactional partners in the instructional project of the riding lesson.

### 1.2. Horse Riding and Horse-Riding Instruction

Riding is an act of interspecies communication. Dashper [18,19] describes the complexities of the purely embodied interaction between horse and rider, which she and others have conceptualized as “equestrian feel” (see also [20]). A number of writers, including the authors of [20,21,22], have explored how riding teachers communicate this feel to their students. Maw [23] details how riding lessons can have a horse focus, a rider focus, or a partnership focus. The latter implies that the rider learns to listen to and communicate with the horse as a partner. Some riders have been shown to conceptualize riding as a “shared action” and horses as “equal partners” ([24], p. 698). However, to our knowledge, there are few studies that focus on the three-way interaction between riding teacher, rider, and horse. Dashper [21] emphasizes that too little attention has been paid to the teacher’s role in the horse-riding lesson triad, but this is beginning to change as several recent studies have focused on riding teachers’ communication [25,26,27,28,29,30,31]. There are even fewer interaction-focused studies that focus on the role of the horse, as highlighted by Zetterqvist Blokhuis [22,32]. Zetterqvist Blokhuis shows that riders play a role as the horses’ guardians during lessons with dressage trainers, making sure the horses are not pushed too hard during different exercises. In contrast, Lundesjö Kvart [33] shows that, unlike the elite equestrian trainers in Zetterqvist Blokhuis’ studies, teachers at riding schools often take on the role of horses’ “interpreters”, ensuring that riders learn to listen to horses’ subtle cues. The riding teachers who were interviewed for Lundesjö Kvart’s study also consider the horses to be their partners in instruction and call them “colleagues”. In light of debates related to horse welfare, riding, and the equestrian world’s social license to operate [34], we argue that delving deeper into how riding teachers and riders listen to horses’ signals during the education of riders will contribute knowledge that increases horses’ agency when interacting with humans.

## 2. Materials and Methods

The data for this study come from three corpora of horse-riding lessons in Sweden, the United Kingdom, and Germany. The Swedish data consist of 40 weekly lessons by four riding teachers (10 lessons each) at different riding schools in Sweden. Individual lessons lasted between 40 and 60 min. Overall, the Swedish corpus amounts to approximately 33 h. The lessons were recorded in 2018 and involved one student per lesson. The horses were riding school horses at an intermediate level, and each student rode the same horse in every lesson. The students had ridden for several years and can be considered relatively skilled riders. The teachers were all educated and experienced riding teachers that had worked at riding schools for at least five years. The teacher–student–horse triads knew each other well, as they had had lessons in these constellations for a long time. Two cameras were used for all recordings: one on the teacher’s head and one capturing the rider and teacher from a corner of the riding arena.

The UK data come from a collection of 16 private riding lessons (dressage, jumping) recorded at varying locations (private yards and riding schools) in the North of England in 2022. The participants are six riding coaches with qualifications from the British Horse Society and 15 private clients and their horses (one pair was recorded twice). A total of 14 students rode at an intermediate or advanced level; one was a relative beginner. All horses were educated to a basic level or beyond and were six years or older in age. Most horses were privately “owned” and cared for by their riders. In two lessons, students rode horses “owned” by a riding school. All human–human–horse triads knew each other well, with the teachers having taught the human–horse combinations repeatedly: in the case of the beginner and the riding school horse, they had done so for several lessons; in the case of some of the private students, teachers had taught the human clients for up to three decades. The UK corpus is approximately 9 h in length; individual lessons lasted between 18 and 48 min. One camera was used capturing all three participants where possible but always the horse and rider. All participants in the Swedish and UK corpus gave written consent for their anonymized data to be disseminated for research purposes.

The German data come from a corpus of approximately 14 h of publicly available recordings of horse-riding lessons on YouTube. This corpus was collected in 2022, with individual videos dating from 2012 to 2019. It includes clinics that are taught in front of an audience as well as private riding lessons. Riders appear to be at an intermediate or advanced level. Some of the horses, especially those in the public masterclasses, are young and in training; the youngest is four years old. All human–horse pairings appear to be established, but in the case of clinics, the visiting riding coaches did not know the riders or horses beforehand. Where possible, consent was sought from content creators and uploaders.

Our transcription follows GAT2 [35] for verbal interaction, with the exception that intonation units are only delimitated by the punctuation marks that indicate phrase-final pitch, not by individual lines. The transcription of embodied actions follows Mondada [36,37]. The transcript notations are provided in Appendix A. It is important to note that the transcripts are limited to features that are relevant to our analysis of each instructional interaction. Where possible, images of the most relevant embodied actions are provided. Of course, the data contain many embodied actions and other events that were not transcribed, as transcripts are always “unavoidably incomplete” [38]. We acknowledge that transcripts are twice-removed representations of the original events and must not be treated as data in themselves [39].

Analytically, this study aligns with Conversation Analysis [15]. It uses naturally occurring data and, through detailed multimodal analysis, tries to unearth the understandings that participants themselves have of each other. This is done by showing how participants respond to other participants’ actions. Horse-riding lessons can pose a challenge in this regard because riders’ actions are mostly invisible, and deliberately so. This is a “members’ problem” ([40], p. 50): riding teachers often have to infer from the way horses move what riders are doing or not doing, because they cannot see, for example, riders’ minimal movements of the reins via their fingers or the pressure they exert with the insides of their calves. This issue is at the heart of our analysis below, as we show that teachers can take their cue from horses’ rather than riders’ actions.

In the following analysis, we use the term (instructional) partner in the sense that the horse is treated as a relevant and acknowledged interactional participant by teacher and rider. By the term “partner”, we do not mean to imply equality of social power. Indeed, many human-only interactions are asymmetric in this regard. There has been much discussion of the role of horses in horse riding, and studies have revealed humans’ complex and at times contradictory characterizations of horses as autonomous agents and equal partners for whom nevertheless the human has responsibility and who therefore need to be controlled [41,42,43]. We use the term “partner” in an interactional sense, in that the horses in our data are treated as equally consequential co-participants and are thus attributed a degree of agency. This conceptualization is irrespective of whether they *have* agency or equality in any empirical sense.

The phenomenon we explore here is ubiquitous throughout our data (see Section 3). Importantly, our concern here is not with frequency but with a conceptualization of horse-led learnables as instances of interspecies interaction, and, specifically, as interspecies learning events. From the many occurrences of horse-led learnables in our data, we have chosen examples that are clear and comparatively accessible to a non-equestrian readership. However, we acknowledge that these data are not as easily understood as everyday human-only talk. This is in part because they include discipline-specific concepts and terminology, which we explain where necessary, and in part because they involve non-verbal communication with another species.

## 3. Emerging Learnables

In many instruction settings for embodied skills, teachers work with a combination of a lesson plan and spontaneous responses to what their pupils present, request, or require on the day. We refer to the process by which an individual lesson agenda comes about as *the emergence of learnables*. In horse-riding lessons, learnables can be, for example, learning how to ask a horse to transition from walk to trot at a near-beginner level or learning how to best jump a combination of fences at an intermediate level. Importantly, we are not interested in these as abstract skills of the horse–rider pair but as instructional concerns that emerge from the local, moment-by-moment interaction between horses, riders, and teachers. Learnables are different from exercises, which are specific tasks that horse and rider are asked to perform, typically in the pursuit of a learnable. New learnables can emerge from exercises and vice versa.

More broadly, learnables can come about in a variety of ways. Majlesi and Broth’s [44] study of second language learning reveals that learnables can emerge in an unplanned way from events or objects within the classroom setting. Zemel and Koschmann [2] show that in surgical training, learnables emerge through instructors’ demonstrations of requested actions as well as trainees’ enactments of them. In Reed and Szczepek Reed’s [45] research on music masterclasses, where “masters” usually do not know their students beforehand, learnables are found to emerge from masters’ displayed expertise, students’ musical performances, and verbal feedback from student-performers or the audience.

In our data regarding horse-riding lessons, we find that learnables can emerge in two basic ways. One is from a pre-instruction discussion between teacher and rider *prior to an instruction sequence*. At the start of the lesson, this can entail looking back over the previous lesson and/or the students’ riding since then and can involve planning for the lesson ahead. Mid-lesson it can entail discussion of the lesson so far and making plans for the upcoming instruction sequence. All our recorded lessons contain such interactions. Importantly, they take place while horse, rider, and teacher are not engaged in concurrent teaching and riding. During pre-instruction sequences, the participants either stand, or horse and rider walk freely on a long rein, that is, they are not physically in a position to perform instructions.

Secondly, learnables can emerge *during an in-progress instruction sequence*, for example, from local events during an ongoing exercise or from the actions of teachers, riders, or horses. In this article, we are interested in learnables that emerge during instruction sequences and from the horse. Every lesson in the data set included many such cases; in most recordings, they are in the majority. This may be in part occasioned by the competence level of our recoded riders. While beginner lessons can focus more on the rider’s body, advanced lessons are often much more concerned with how the horse is going. Since a large part of riding involves negotiating with the horse about how they move their body, riding instruction mostly focuses on how the horse moves. Therefore, learnables often emerge from horses’ bodily actions.

*Horse-led learnables* are verbally raised by the human participants (teacher or rider), who treat them as initiations of new courses of action. Our analysis below focuses on such instances, that is, on instruction sequences that develop or change trajectory in response to the horse’s bodily actions. We argue that by taking their cue from horses, riding teachers treat them as instructional partners and as co-teachers of their riding pupils. In using the term “horse-led”, we are not implying that horses “deliberately” decide to steer the lesson in a certain direction. We simply mean that horses’ actions can initiate new sequences of action.

A representative first example shows how a new learnable can emerge from the horse’s actions. The extract comes from a private lesson in a Swedish riding school. Before the transcribed section, horse and rider are walking on a circle in front of the teacher, who is giving instructions on how to work the horse in canter, which is the horse’s three-beat gait (in contrast to the four-beat walk and the two-beat trot). The current learnable is to canter with energy and “collection”, that is, with the horse carrying more of their weight on their hind legs rather than in front. However, in response to the way the horse “runs off” (line 13) into the canter, the teacher turns her focus from *how the canter should be ridden* (the original learnable) to *how the transition from walk to canter should be ridden* (a new learnable) and later to *how a circle should be ridden* (another new learnable). Both changes to the teaching agenda are based on the horse’s movements.

Extract (1) SWE Rider L Lesson 2

Learnables emerge from the horse’s actions (T=teacher, R=rider, H=Horse) 
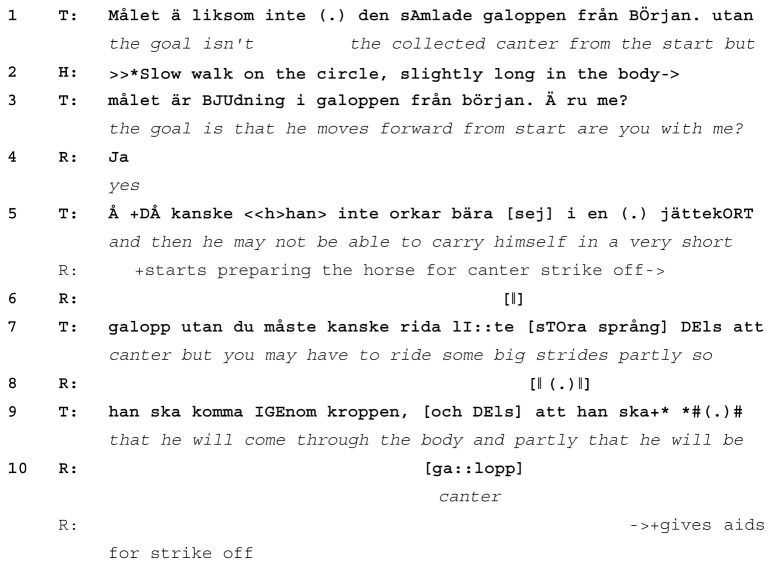
 
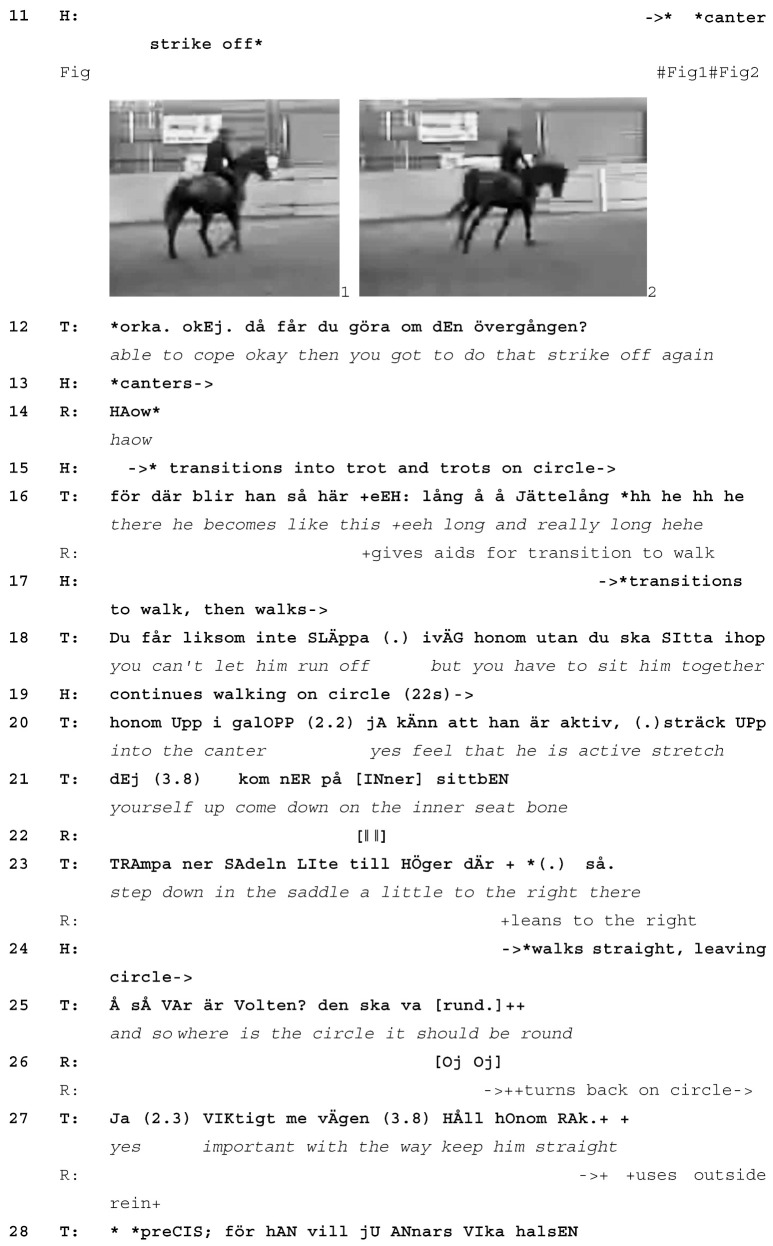
 
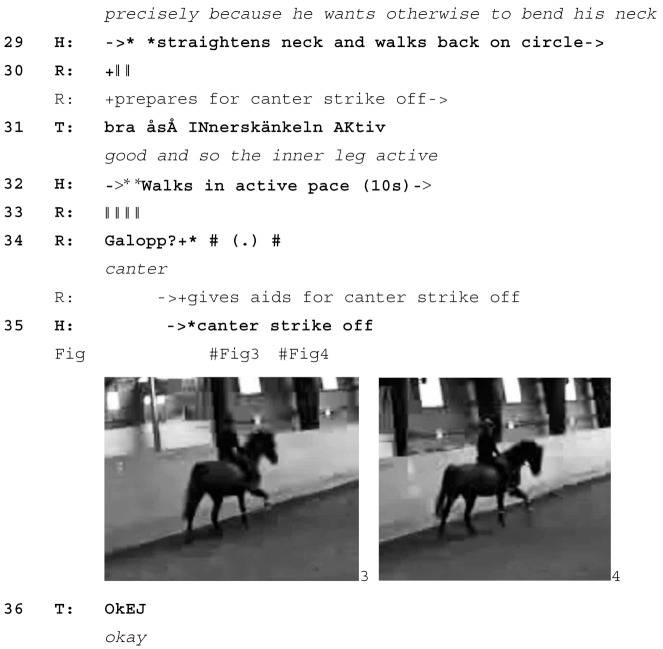


While the teacher is giving extended instructions on how the rider should make the canter forward-going and collected, the rider asks the horse to strike off into canter (line 10). As soon as she does so, the teacher interrupts herself with *okay* (line 12) and immediately asks the rider to come back to walk and do the strike off again, explaining that this is due to the horse having become *really long* (line 16) and having *run off* (line 18). The teacher’s comments refer to the horse stretching out the neck too far, which results in too much weight on the front legs (line 11, Images 1 and 2). Since the original learnable was “collection” in canter, that is, a canter where the horse carries more weight on their hind legs, starting out with too much weight on the front legs is undesired. By addressing the issue of the strike off as one related to the horse (becoming too long, running off) rather than the rider, the teacher shows that she is taking her cue from the horse’s actions and treats them as initiating a new learnable: how to do a canter strike off without letting the horse become too long. This reduction in focus from a larger exercise (“how to do forward and collected canter”) to one that is more basic and smaller in scope (“how to get the horse into canter”) is typical of learnables that emerge during an instruction sequence.

The teacher continues to give instructions on how the rider should sit in the saddle when yet another learnable emerges, again initiated by the horse’s actions (line 24). While still in walk, the horse does not follow the circle track but instead bends his neck and walks straight on. Once again, the teacher interrupts her ongoing instruction on how to sit in the saddle for strike off (line 25) with *where* and uses the horse’s actions to introduce a new learnable (how to keep the horse on a circle, lines 25–31). Here, again, the teacher takes her cue from the horse. When horse and rider follow the circle track again, she returns to the canter strike off as the current learnable, which is eventually achieved when horse and rider make a new strike off with more collection (line 35, Images 3 and 4), visible in the horse’s shorter and higher neck carriage.

The example shows a type of instruction sequence that will be familiar to many riders. The teacher decides what horse and rider should work on; here, it is the collected canter. However, as horse and rider set out to comply with the instruction, the horse’s movements draw the attention of the teacher and inspire a new focus; here, how to transition from walk to canter. The new learnable is once again re-directed when the teacher responds to another action from the horse, namely the horse walking straight instead of on the circle. This “Russian doll” structure of a learnable within a learnable within a learnable originates from the horse’s movements. We argue that new learnables emerge as the teacher responds to the horse and adjusts her pedagogical focus in line with his or her actions. The rider’s actions are of course important in this context, since it is the negotiation between rider and horse that the lesson is concerned with. However, the emergence of these new learnables is led by and attributed to the horse.

## 4. Results: Horses as Instructional Partners

The following sections present interaction analyses of how horses’ actions can initiate learnables that humans take up and respond to. The data show that this can occur in three ways. Firstly, learnables can emerge from *horses’ actions that display their overall internal state* (e.g., liveliness or anxiety) or *physical abilities and preferences* (e.g., preferring moving in one direction over another). Secondly, learnables emerge from *horses’ actions that directly respond to the local contingencies* of the instruction environment (e.g., a flight response to an unknown object). Thirdly, they can emerge from *horses’ actions that directly respond to riders’ actions* (e.g., an incorrect use of the reins or legs). In this way, horses become instructional partners in the interactional project of the horse-riding lesson. In the third case, they are not only framed as instructional but also as diagnostic partners by drawing attention to the rider’s incorrect actions through their responses.

### 4.1. Horse-Led Learnables: Horses Display Their Internal or Physical State

Horses can influence the course of a riding lesson through behaviour that pre-dates a specific instruction sequence and displays an internal or physical state. For example, horses may come to the lesson with high or low energy or their skeletal confirmation may affect how they can perform certain exercises. In these cases, teachers can take their cues from the horse and turn working with these behaviours into learnables for rider and horse. The following two extracts are clear cases.

The extract below comes from the UK corpus and shows a private lesson on the client’s own horse. The rider is a horse racing jockey who rides a racehorse in a lesson where both are learning basic dressage, that is, a different way of riding to what they are used to. The horse is full of energy and finds it hard to concentrate on what the rider is asking. The horse’s energy level is topicalized several times throughout the lesson. For example, 2 min 13 s before the transcribed section, the teacher tells the rider: “With a horse like him that’s so stressy, it’s such a skill to be able to sit so quietly and not react to it … and you’re doing a really good job of that”. Immediately prior to the extract, horse and rider have been trotting (the horse’s two-beat movement) with a view to start cantering (the faster three-beat movement), which is the next planned learnable. Given the horse’s displayed state of mind, the canter has the potential to get too fast or out of control. At lines 1–2, the teacher gives the directive to canter. Horse and rider are on a circle of approx. 20 m diameter around the teacher. The extract shows how the horse’s actions first lead the teacher to delay and later abandon the planned learnable of *work in canter* and how subsequently a new learnable, *relaxed trot*, emerges instead.

Extract (2) ENG 22020605 Lesson 2

Learnable emerges from the horse displaying his internal state (T=teacher, R=rider, H=Horse) 
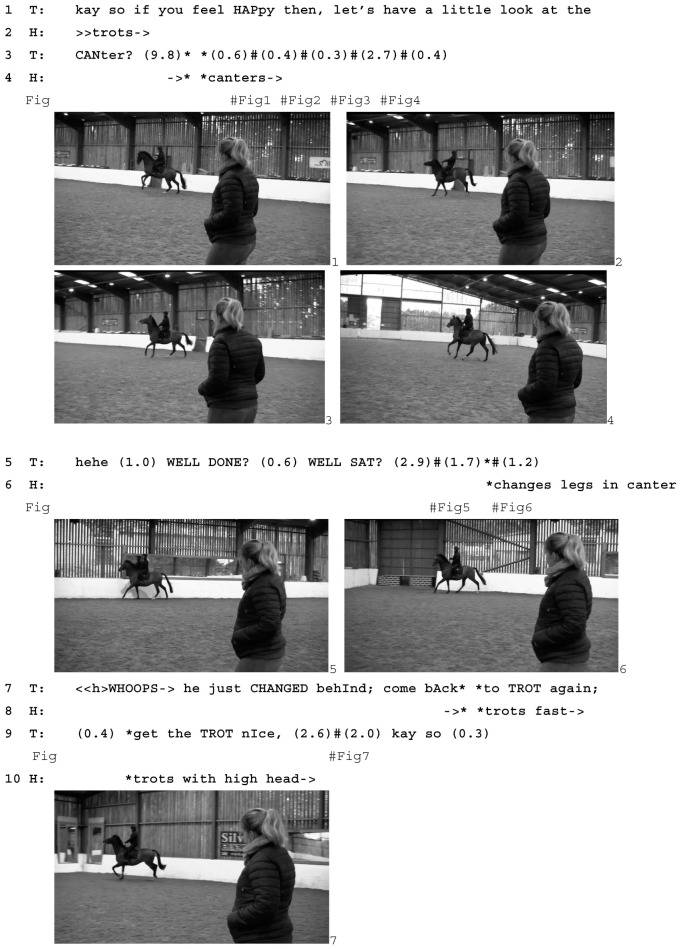
 
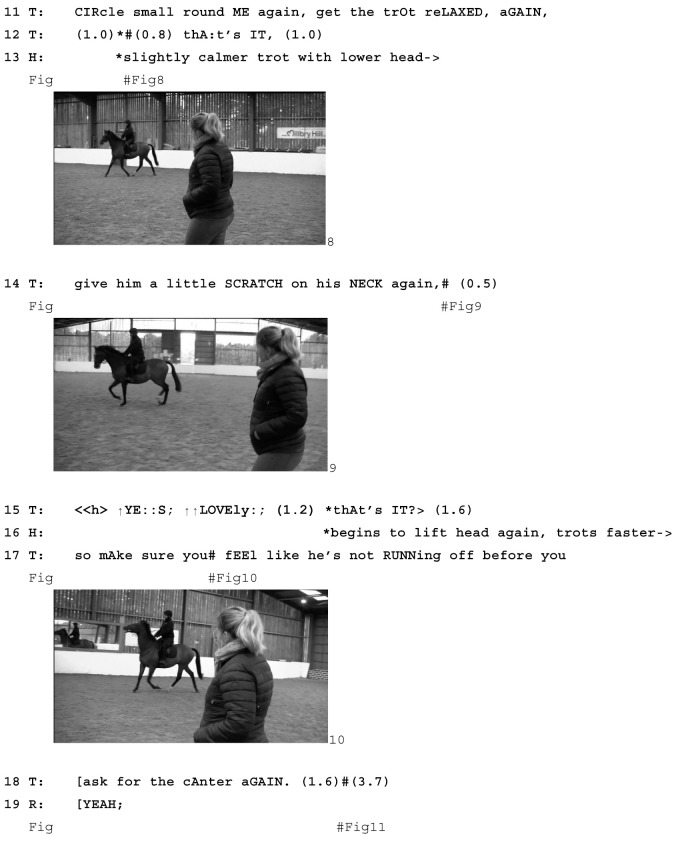
 
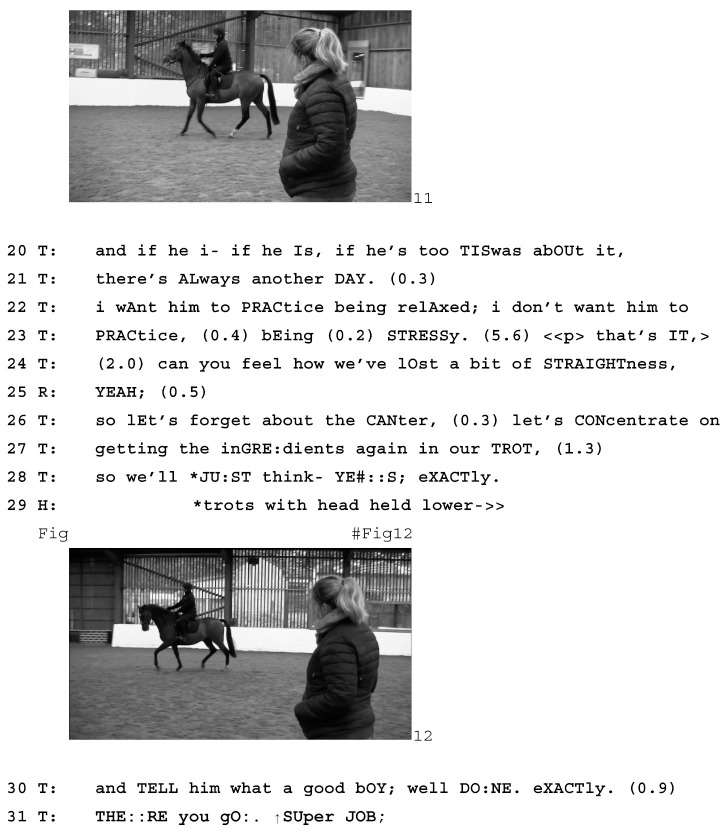


After the teacher has given the directive to canter, that is, the directive to start the planned learnable, the rider takes approximately 10 s before giving the horse the relevant aids, presumably waiting for the right moment to ask for canter. When he does, the horse responds with a lot of energy (Image 1–3) and, after a few strides, swings his hind quarters into the circle (Image 4). The rider’s task at this point is not only to keep the horse at a reasonable speed but also to keep him on the circle rather than shooting off to the other side of the arena. As the rider steers the horse back onto the circle, the teacher laughs and gives a positive assessment (line 5). She makes no mention of the horse’s actions but instead treats them as a minor source of amusement and makes verbal reference only to the rider’s way of dealing with them (*well sat*).

As the horse continues to canter in a fast and excited manner, he changes the order in which he puts his hindlegs on the ground. Image 5 shows “normal” canter, as the inside hind leg comes forward as the outside front leg comes back. Image 6 shows the moment after he has changed his hind legs around, with the *outside* hind leg forward as the outside front leg comes back. The teacher treats this as a trouble source: *whoops he just changed behind come back to trot again* (line 7). In this instance, changing legs is another sign of the horse’s overall excitement. By responding with *whoops,* the teacher treats the horse’s leg change as nobody’s fault. *Whoops* is what Goffman [46] calls a “spill cry” (p. 101), which “defines the event as a mere accident” (p. 102).

In asking the rider to bring the horse back to trot, the teacher abandons the exercise. Initially, she appears to do so only temporarily. Once the horse has returned to trot, he is moving fast and impatiently, which involves him holding his head very high (Image 7). The teacher asks the rider to *get the trot nice* (line 9) and *get the trot relaxed* (line 11), and the horse gradually relaxes and lowers his head (Image 8, 9). However, he soon starts throwing his head up again (Image 10). At this point, the teacher is still oriented to another attempt at canter (lines 17–18); however, in response to the horse’s continued tense and energetic trot (Image 11), she prepares for abandoning the task altogether (*if he’s too tiswas about it there’s always another day*, lines 20–21). Soon afterwards, she points out a loss of *straightness* in the horse’s way of moving (line 24), a sign of him now not being balanced enough to transition to canter in a controlled way. This finally leads her to abandon the exercise completely: *so let’s forget about the canter* (line 26). Instead, she initiates a new learnable, a correctly ridden trot (lines 26–27), which horse and rider achieve quickly (Image 12). This is met with closing assessments from the teacher (lines 28, 30–31).

We argue that by taking her cue from the horse, the riding teacher treats his actions as consequential for her instruction. However, rather than framing the horse as accountable for the failure of the exercise, she ascribes to him the agency of an instructional partner who determines the course of the lesson. She does this initially by making no reference to his actions—which are in fact rather disruptive to her initial plans for this part of the lesson—and by treating them instead as a given; specifically, she treats them as mildly amusing and accidental. Once the horse has initiated a break with the current exercise and returned to trot, *working in trot* becomes the new—initially only temporary—learnable. Once the horse makes it clear that cantering calmly is not something he is able to do at this time, *relaxation in trot* is established as a new learnable and is jointly achieved by horse and rider.

The extract shows that framing the horse as an instructional partner involves both an attribution and a disattribution of agency: by treating the horse’s actions as an externally given factor, the teacher treats them as unintentional and thus the horse as unaccountable. By making her in-progress instruction dependent on how the horse acts, she attributes to him the agency and power to determine their three-way interaction.

The following example from a Swedish private riding lesson at a riding school shows another case of a horse-led learnable. Early on in the lesson and 17 min prior to the transcribed section, the teacher comments on the horse’s physical straightness as noticeable (*now he at least walks straight*). This refers to the fact that, like many horses, he prefers to bend more to one side than another when moving. The teacher frames the horse’s lack of straightness as somewhat problematic. It may be the case that, since the horse is “owned” by the riding school (rather than the rider), the teacher knows the horse well and is aware of the problem. At this point, the teacher also announces the planned exercise and subsequent learnable: *we start with a little warm-up work for his straightness and then I think we can work on simple changes*. The term “simple change” refers to a change of direction in canter that involves a transition into walk and back into canter but in a new direction. When changing direction in canter, horses typically change the order in which they place their legs on the ground, with the inside front leg “leading” the stride.

The important point here is that, while straightness is identified as an early focus for the warm-up, the main emphasis of the lesson is planned to be on something else (simple changes). A potential reason is that the lesson takes place at a riding school, where the main purpose of the lesson is not to work on the horse’s problems but to educate the rider. In line with this plan, 4 min 22 s before the transcribed section the teacher asks the rider to start work in canter and, in preparation for simple changes, instructs her to canter on a straight line towards her, two meters in from the track. As horse and rider do so, it emerges that the horse is still not straight enough in canter. This discovery leads the teacher to change her focus from *simple changes* to a new learnable of *keeping the horse straight*. At the start of the transcribed section, the rider is cantering towards the teacher.

Extract (3) SWE Rider L Lesson 6

Learnable emerges from the horse displaying his physical state (T=teacher, R=rider, H=Horse) 
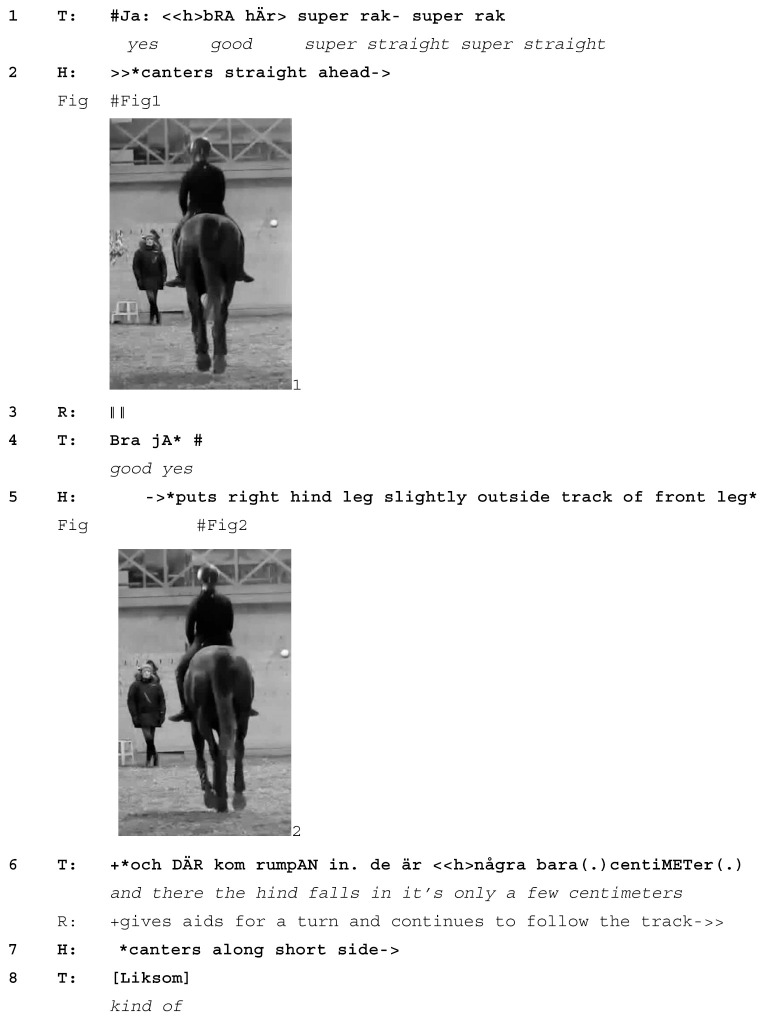
 
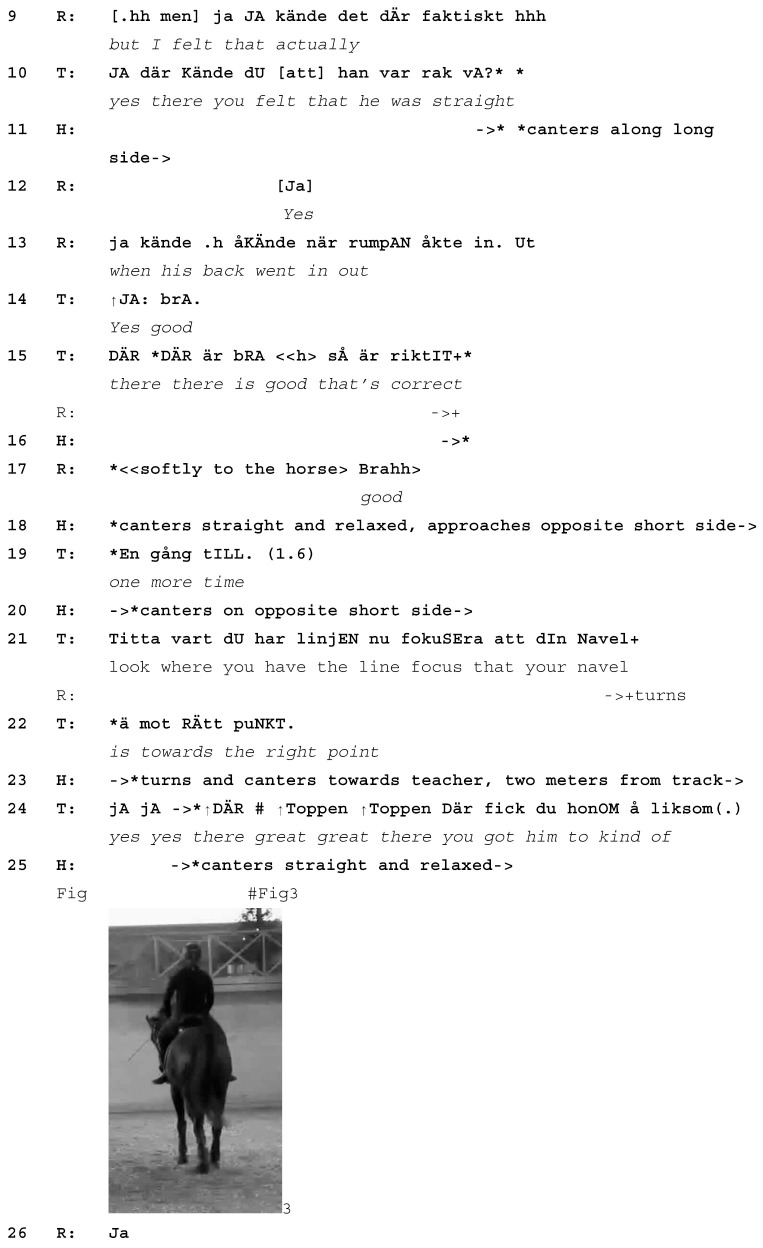
 
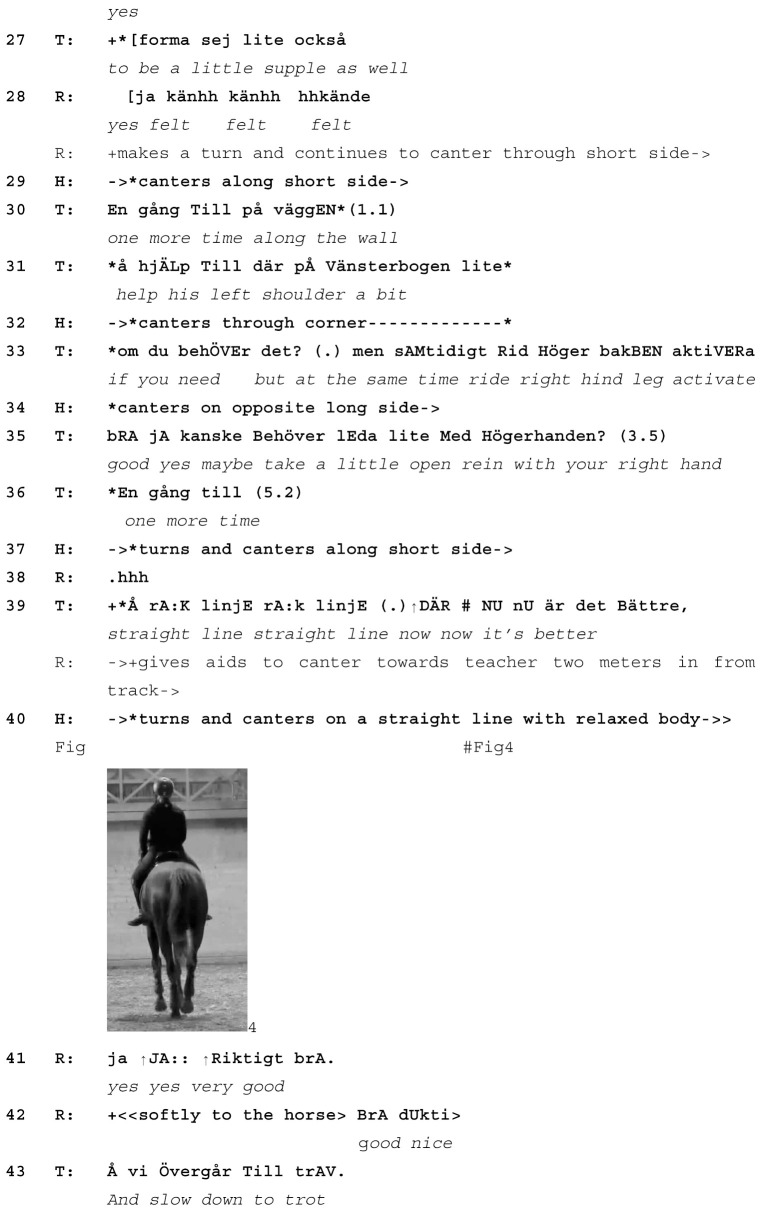


Initially, the teacher praises rider and horse for being *super straight* (line 1, Image 1). But after a few straight strides towards the teacher, the horse put his right hind leg outside the track created by the front legs (line 5, Image 2) and the teacher promptly responds (*and there the hind falls in*, line 6) with emphasis on the word *there*. This gives the rider a chance to feel the horse’s action in her body. The teacher follows up on this by focusing on how the rider *experiences the horse’s straightness* (lines 10–14), which now develops into the new horse-led learnable. The teacher then instructs the rider to repeat the same exercise three more times (line 19, 30 and 36). Each time horse and rider approach the teacher head on, the teacher assesses the straightness of the horse’s body (e.g., line 24, Image 3 and line 39, Image 4). The teacher thus draws the rider’s attention to the horse’s actions, treating the horse as an interactional partner in the work. The rider praises the horse (*good*, line 17, *good nice*, line 42) and in doing so also acknowledges the horse’s involvement in performing the exercise. In addition, the rider’s praise displays understanding of the horse’s physical state and his difficulty in cantering in a straight line.

During a short break after the end of the extract, the teacher and rider discuss the exercise. The rider comments that this had been the first time she clearly felt the improvement and subsequent loss of straightness in the horse as he responded to her communication but then lost the strength to maintain the straight body posture. Here, the horse’s actions (shown in line 2/Image 1, line 5/Image 2, line 25/Image 3 and line 40/Image 4) are interpreted and verbalized by both teacher and rider, thus ascribing to the horse the role of a co-teacher based on his physical responses. Following this discussion, the teacher asks horse and rider to do the same exercise (straightness in canter) in the other direction and subsequently introduces a new learnable (*turn on the quarter in walk*), having decided that the horse has done enough cantering. Thus, the pre-instruction plan to work on simple changes is abandoned in response to, and out of respect for, the horse’s display of his physical state.

Extracts (2) and (3) show how horses’ displays of pre-existing internal and physical states are treated as initiations of new learnables. In the following, a learnable emerges from the horse’s response to the local situation.

### 4.2. Horse-Led Learnables: Horses Respond to the Local Instruction Environment

A second way in which horses can bring about new learnables is by responding to the local instructional context. The following example shows how a riding teacher treats such a response as initiating a new learnable. In this extract from the German YouTube corpus, a young horse is being ridden by an advanced rider in a public dressage masterclass taught by a well-known equestrian and coach. The transcribed section occurs early on in the lesson as horse and rider are trotting around the arena. The audience is seated on two sides: along a short side on a tiered stand and along a long side at ground level, where there are also other objects, including three parked cars. Every time the horse has to go down this long side, she looks uneasily at it. At 26 s before the start of the transcribed section, the rider guides the horse on a circle for the first time, having so far only ridden along the outside track. This means that they have to come across the middle of the arena and approach the challenging long side head-on. When the horse is half-way across, she jumps at a noise and rears up. The rider calms her quickly, and the pair soon return to trotting around the circle. At that point, the teacher does not give the rider advice on how to deal with the situation but comments on horses’ reactions more generally. Immediately afterwards, at the start of the transcribed extract, the teacher turns her attention to the rider’s hands, which are held very high. Horse and rider are now trotting around the circle for the second time and are about to ride directly towards the audience again.

Extract (4) GER 20151013 Lesson 1

Learnable emerges from the horse responding to the environment (T=teacher, R=rider, H=Horse) 
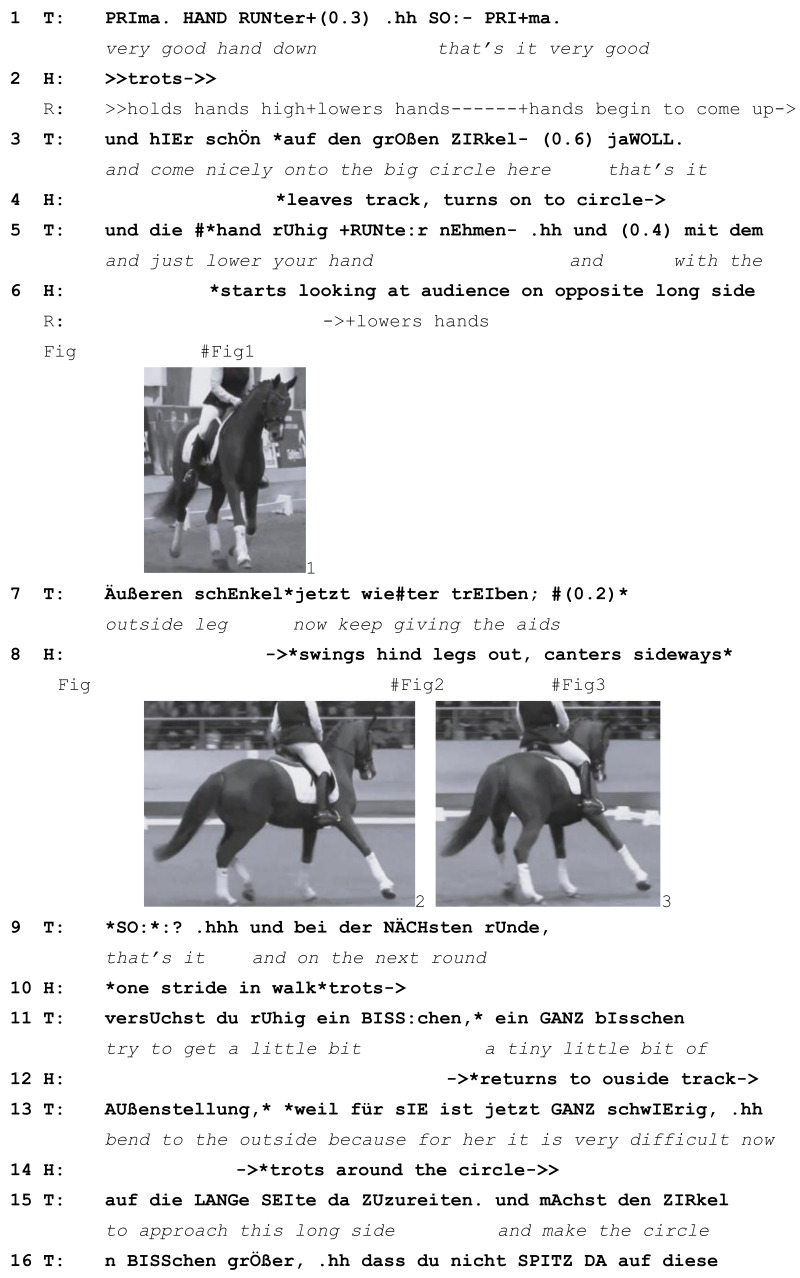
 
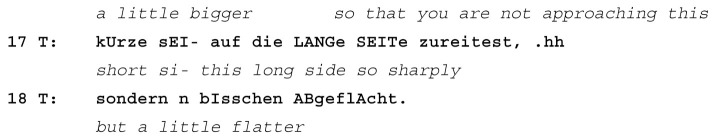


In line 1, the teacher asks the rider to lower her hands. The correct positioning of the hands will continue to be a learnable throughout the session, with 29 directives focused on lowering the hands over the course of the 25 min lesson. This is the first instance. Immediately upon receiving the directive, the rider lowers her hands. However, soon afterwards, she begins to raise them again (line 2). As horse and rider turn on to the circle, the teacher gives another directive to lower the hands (line 5). While she delivers this directive, the horse starts raising her head and looks at the audience, who are now once again seated directly in front of her (Image 1). In response to this, the teacher turns her attention away from the rider’s hands. Dealing with the horse’s response to the audience becomes the new learnable. The teacher gives the directive to keep using the outside leg, that is, to guide the horse to stay straight—most likely the teacher can foresee that the horse is about to swing her hindquarters out, which she indeed does soon afterwards (line 8, Image 2) and which the rider’s outside leg can contain. The horse swings around slightly and starts cantering sideways (Image 3). Very quickly, the rider calms her, and the horse takes one step in walk before trotting past the audience and around the circle. The teacher gives a positive assessment of the rider’s success at containing the situation (*that’s it*, line 9) and then constructs a learnable from the horse’s response to the situation. She asks the rider to help the horse and make the circle bigger in future so that she does not have to approach the audience head-on (lines 11–18).

Once again, a teacher abandons a previous learnable (*correct positioning of the hands*) and responds to actions by the horse, which initiate a new learnable (*riding through a challenge*). The horse is again treated as prompting a new learnable that otherwise would not have arisen and thus as a partner in the instruction sequence. Like the coach in extract (2), this teacher does not mention the horse’s behaviour explicitly but only the rider’s success in handling it. As above, no fault or intention is ascribed to the horse. Instead, her actions are explicitly accepted and explained (*for her it is very difficult now to approach this long side*, lines 13, 15), and a learnable is developed from them.

Extract (4) demonstrates that horses can bring about new learnables by responding to the local specifics of the instructional sequence. Our final section shows how new learnables can emerge from horses’ responses to riders’ communication with them.

### 4.3. Horse-Led Learnables: Horses Respond to Riders’ Actions

Finally, new learnables can be initiated by horses’ responses to riders’ actions. For example, a rider’s incorrect leg position or weight distribution can lead to the horse moving in a way that was not intended by the rider. As the horse responds to the rider’s embodied aids, they bring them to the teacher’s attention. Here, in addition to being treated as *instructional* partners, horses are also framed as *diagnostic* partners, as teachers draw on horses’ actions as a resource for showing what riders did correctly or, more often, incorrectly. This kind of horse involvement in the riding lesson is the most interesting with regard to interspecies pragmatics, as it most clearly shows that horses’ acts are treated as “actions” in response to human actions, rather than “behaviour” that occurs irrespective of interaction with the humans. We present two examples that reveal some of the nuances of these sequence types.

In the following example from a private lesson in a Swedish riding school, the rider is working in canter. A new exercise is introduced in that they are being asked to ride a shallow loop, that is, to leave the track by a few meters at the long side of the arena and then return to it. At 11 s before the extract begins, the teacher instructs the rider to sit still in the saddle and let the horse canter on his own (*don’t over-ride now, sit still, he must be able to canter without you interfering*). By “over-riding” and “interfering” the teacher refers to the rider being too active and possibly out of balance, which can cause them to give unintended aids to the horse. At the start of the extract, the rider is beginning to ride the shallow loop in canter.

Extract (5) SWE Rider G Lesson 10

Learnable emerges from the horse responding to the rider’s unintentional action (T=teacher, R=rider, H=Horse) 
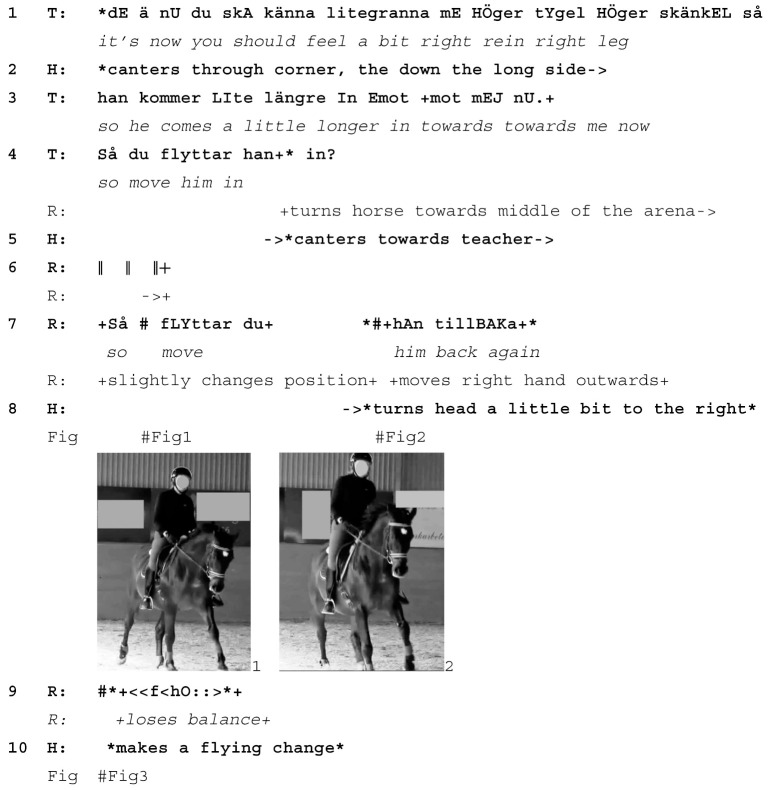
 
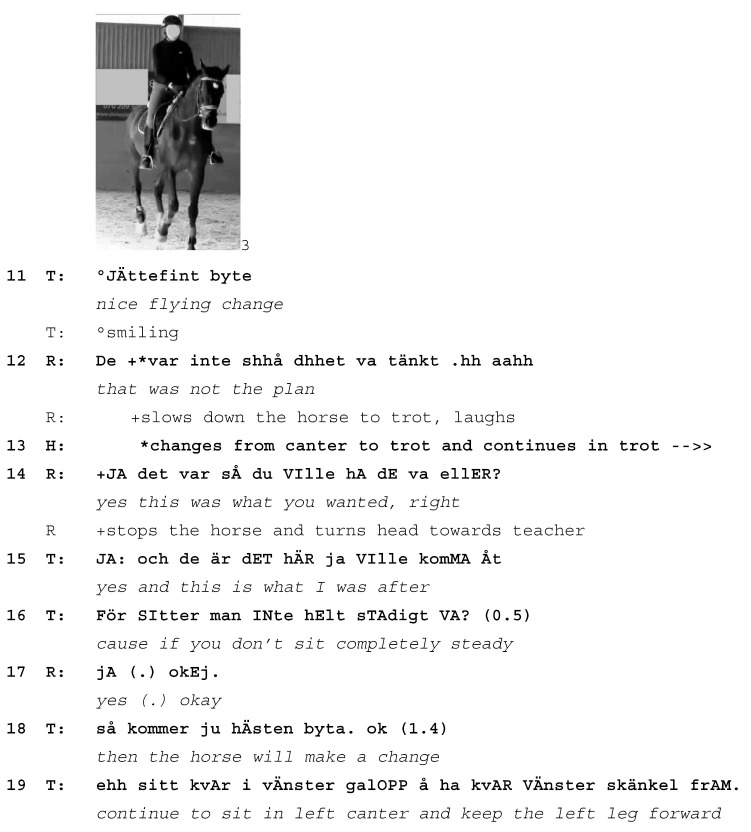


The teacher instructs the rider first to move the horse towards the middle (line 3) and then turn back to the track (line 7). The rider follows the teacher’s instruction, enacting the limited time window and “instructional space” [27] that is typical of riding lessons, where learners are required to act quickly.

In response to the instruction to move back to the track, the rider pulls the right rein and turns her gaze towards the track (line 7, Image 1 and 2), which causes the horse to perform a flying change (line 10, Image 3). This means he changes direction in canter with one single canter stride. He interprets the rider’s head and body turn towards the track and the resulting shift in her bodyweight as a signal to make a flying change and responds accordingly. The rider exclaims *hoo* (line 9)), which is a “spill cry” [46] similar to that of the teacher in extract (2), and which displays the rider’s awareness of an unintended miscommunication.

Teacher and rider treat the incident with smiles and laughter (lines 11–14): the flying change is in fact a rather difficult exercise when it is intended. The teacher goes on to explain that she had expected this to happen (line 15) and that the mistake occurred due to the rider not sitting *completely steady* (line 16). She instructs the rider how to sit correctly (line 19). In having designed the exercise such that an incorrect seating position by the rider can bring about the “wrong” response in the horse, the teacher treats the horse not only as a co-teacher but also as a diagnostic partner. As a result, the learnable emerges through an instructional collaboration between teacher and horse.

Our last example shows how an initially simple correction of the rider’s actions becomes a fully developed learnable following the horse’s response to the rider. At the start of this extract from the German YouTube corpus, the exercise is to ride smooth transitions from trot to walk and back to trot. Horse and rider have been asked to perform these transitions repeatedly; the learnable here is to help the horse balance herself. The horse is 5 years old and still learning. The rider is at an advanced level, and the lesson is a public masterclass with another famous equestrian and coach (a different coach from the one in Extract 4). The transitions are initially judged by the teacher to be too “abrupt” and not balanced enough. Prior to the transcribed extract, horse and rider do eight trot-walk-trot transitions during which the teacher talks about helping the young horse stay balanced during the transitions. The transcript shows the nineth attempt (lines 1–7), during which the horse again loses her balance, which can be seen by her dropping her head (line 4) in an attempt to regain balance. Nevertheless, this attempt is the best so far and the teacher asks the rider to praise the horse (line 6).

Extract (6) GER 20160425 Lesson 1

Learnable emerges from the horse responding to the rider’s incorrect practice (T=teacher, R=rider, H=Horse) 
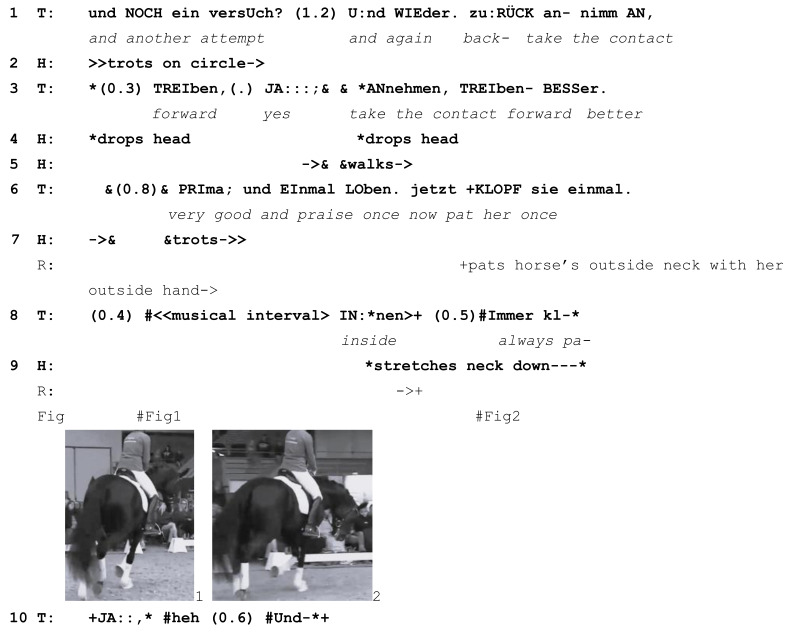
 
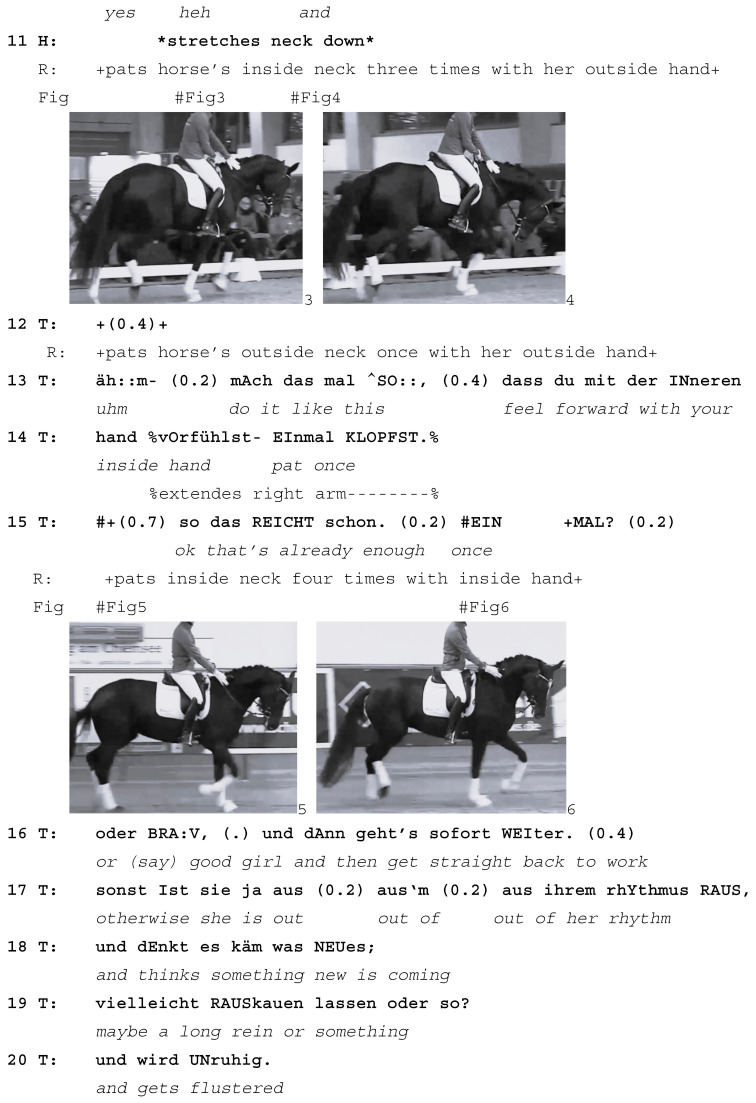


The rider is being asked to pat the horse’s neck as a sign of praise (line 6). She pats the left side of the horse’s neck with her left hand (line 7; Image 1); left is on the outside of the arena at this moment. This is met with almost simultaneous responses from both the teacher and the horse. The teacher corrects the rider by asking her to pat the horse on the inside of the neck instead, a turn that is aborted (*inside always pa-*, line 8) in response to the horse’s simultaneous reaction to the rider’s patting: while the teacher is still speaking, the horse takes the longer outside rein that is now slack due to the rider’s patting and lowers her head, stretching her neck down and to the right, where the rein is still short (Image 2). The teacher aborts her correction turn and receipts the horse’s stretching of her head with a hesitating, elongated *yes* with rising pitch (line 10). This response verbally receipts the rider’s and horse’s actions, possibly implying that the horse’s response was something that the teacher expected to happen. Prosodically, the rising *yes* projects that more may need to be said.

In response to the teacher’s correction to pat the horse *inside*, the rider now pats the inside of the horse’s neck three times; however, she does so with her *outside* hand, reaching across the horse’s withers to do so (line 11, Image 3). Almost immediately, the horse once again stretches her neck down and to the right in response to the looser outside rein that results from this way of patting (line 11, Image 4). On both occasions, her neck stretching results in a loss of balance and rhythm in her trot.

The teacher produces a single laughter particle (line 10) and waits for the rider to finish patting the horse (line 12) before she corrects her again. This time, she offers both a verbal explanation and—presumably—an embodied demonstration (*do it like this*, line 13, not on camera) of what she is asking the rider to do, which is to pat the horse *once* on the *inside* of her neck with her *inside* hand (lines 13–14). In response, the rider pats the horse four times (instead of once) but with the requested inside hand (lines 15). This time, the horse’s head and neck stay up (Images 5 and 6) and she also keeps her rhythm in the trot.

In her subsequent talk, the teacher explains why the horse should always be patted on the inside and only once. In her development of the learnable, she picks up on two of the horse’s prior actions: her loss of rhythm (line 17) and her stretching her head down, which is interpreted as the horse anticipating being ridden on a long rein (lines 18–19). Giving the horse a long rein is typically done at the end of an exercise and means the horse can relax. The outside rein is much more important for the horse’s balance than the inside rein, which explains both the response by the horse (stretching down and thus displaying an interpretation of the longer rein as “we’re finished”) and the teacher (insisting on patting with the inside rein and only briefly, thus not letting go of the all-important outside rein).

The extract shows how the horse’s response to the rider’s actions is picked up by the teacher and turned into a learnable for the rider. The teacher moves away from the earlier learnable, *improving the horse’s balance through trot-walk-trot transitions*, to focus instead on *patting the horse correctly*. What may have been a simple repair sequence is developed into a longer learnable sequence in response to the horse’s neck stretches and resulting loss of rhythm. In this way, the horse’s actions are being treated as initiating a new learnable. The teacher frames the horse as both an instructional and a diagnostic partner: not only is the horse treated as a co-teacher, she also reveals the precise actions that the rider must improve. Here, patting for too long and with the wrong hand releases the crucial outside rein for an extended period, which gives the horse the signal that she is finished for now and can stretch her neck out.

The extracts in this Section have shown how horses’ responses to riders’ signals help diagnose the quality of the rider’s actions and initiate new learnables. Learnables can take the form of diagnostic unpacking, which can be brief or more extended. By treating horses’ responses as consequential for how the lesson progresses, teachers treat them as both instructional and diagnostic partners in the overall project of the horse-riding lesson.

## 5. Discussion

The analysis above makes a case for theorizing interspecies interaction as a genuinely collaborative endeavour. In the same way that interaction between humans is socially built, managed, and negotiated, interaction between humans and horses is jointly achieved via the ongoing work of relating to others, including their past and future behaviour. Specifically, the data show that riders and riding teachers interact with horses in ways that co-construct them as interactional partners in riding instruction. By building instruction sequences on horse actions—which may themselves respond to human actions, or not—riding teachers treat the project of riding instruction as an interspecies collaboration.

Our study underscores a specific challenge that riding teachers face with regard to human and equine needs. While the main purpose of a riding lesson—certainly at a riding school that provides the horses—is to *educate riders*, research has shown that riding teachers also perceive themselves as *horses*’ “*interpreters*” [33] and responsible for their mental and physical wellbeing. Our data reflect different foci in this regard, as instructional sequences can have more focus on horse or rider [23,33], respectively. We speculate that this distinction, in part, relates to whether the horse’s main human guardian is the rider, in which case the horse-rider equipage is treated as a team of two learners, or whether the horse “belongs to” the teacher or their employing riding school, in which case the rider is very much in focus while the horse’s learning happens mostly outside the lesson (for example, by being trained by professional riders). Examples of such a distinction are extracts (2), the jockey and his racehorse learning dressage with a private coach, and (3), the client on a riding school horse. In both cases, horses change the course of the lesson through displays of their internal or physical states. In (2), the focus is on how the rider can help the horse to calm down, i.e., the emphasis is on helping the horse. In contrast, in (3), the focus is on teaching the rider to feel the horse’s body and to correct the horse’s movements through their own. Here, the emphasis is on the rider and their learning journey. Whether a teacher places more focus on the horse or the rider is likely to be influenced by other factors, too. For example, in extracts (4) and (6), we see two famous equestrians who teach advanced riders. Both coaches focus on helping the horse without “owning” or even knowing them, thus arguably adopting a position of authority on all horses.

The above distinction sits alongside the main finding of our study, which is that horses are treated as instructional partners in naturally occurring horse-riding lessons. Our finding supports a broader trend in equestrian research and discourse. It aligns with Wadham’s and Dashper’s [47] multispecies ethnography of horses in tourism, which finds that horses are seen as “co-workers and epistemological partners” (p. 1) in the forestry and trekking industry. Our study also confirms an earlier finding from an interview study by Lundesjö Kvart that “horses are considered workmates, as the riding teachers believe that the horses have an active role during the riding lesson as assistant teachers and they speak of the horses as colleagues” ([33] p. 73). Along similar lines, the managing director of one of Germany’s largest riding schools has argued in a recent interview that the term “teaching horses” (“Lehrpferde”) should be preferred over the more widely used term “school horses” (“Schulpferde”) because it reflects the horses’ role as the riding school’s “most important staff members” [48] (translation by the authors).

Our data reveal that this view of the horse as a riding teacher is *socially enacted* in riding lessons. Horses are not only treated as co-teachers who inspire new learning trajectories but they also become diagnostic partners who “model” [49] the effect that riders’ actions have—or do not have—on them. Using the lens of Conversation Analysis helps us to see and reflect on how horses interpret riders’ embodied actions and to what degree unintentional embodied actions by the rider can be perceived as meaningful by horses. An important aspect of riding lessons is learning not only what cues to give as a rider but also what cues one may be unintentionally giving. An uncomfortable challenge exists with regard to how much unintentional communication can be tolerated when it results in mixed messages and even physical discomfort for the horse. In this context, it is important to consider horses not only as co-teachers but also as co-learners who experience riders’ cues as well as the consequences of different responses to them. (We would like to thank one of the reviewers for making this important point). The human teachers’ interactional work has relevance for the welfare of the horses, as it is their responsibility to ensure that riders understand horses’ actions and responses correctly—as far as this is possible—in each situation. Navigating this challenge relates back to the demand on riding teachers to cater to both riders and horses in parallel. The practices we see in our data show teachers’ ongoing interactional negotiation of this challenge.

## 6. Conclusions

Learning to ride is often referred to as acquiring a certain “feel” for the horse, a process that has been described as “elusive” ([22] p. 107) and “felt and negotiated through intimate body-to-body communication” ([21], p. 88). While much research on equestrian learning is focused on the rider’s feel, our study has considered the three-way interspecies interaction between horse, rider, and riding teacher, and, specifically, the horse’s role in the riding lesson. Interspecies interaction is still a growing field of research, and this study contributes to it by showing how humans interpret horses’ actions and how they treat them as consequential. We still know too little about horses’ minds to make assumptions about their intentions. However, it is an intriguing thought that a horse’s action, such as a flying change (extract 5) or stretching the neck out (extract 6), could be considered a request made by the horse to the human. Whatever we decide to call horses—instructional partners, colleagues, teaching horses—we must listen to their social communication and allow them agency to act in line with their species-specific behaviour when interacting with humans. In this study, we have shown riding teachers’ practices for doing so. Learning from and improving those practices must be an objective for future equestrian researchers and practitioners.

## Data Availability

The raw data supporting the conclusions of this article will be made available by the authors on request where they meet participant consent restrictions.

## Appendix A

Speech transcription is adapted from Selting et al. [35] and the multimodal from Mondada [37].

(.)	micro pause
(1.2)	measured pause
:::	lengthening
ACcent	primary pitch accent
Accent	secondary pitch accent
<<h> >	high pitch register
<<f> >	forte
.h .hh .hhh	in-breath
h hh hhh	out-breath
ǁ	click
[talk	
[talk	overlap

Phrase-final pitch movements:

?	rise-to-high
,	rise-to-mid
-	level
;	fall-to-mid
.	fall-to-low

Embodied actions:

#	Indicates the exact moment at which a screen shot was taken.
**,%%,&&	Embodied actions are delimited between two identical symbols (if the same action continues but in a modified manner, it is not marked as complete but as modified).
*—>	An action continues across subsequent lines.
—>*	An ongoing action comes to completion.
>>	An action started before the transcribed excerpt.
—>>	An action continues beyond the transcribed excerpt.
